# Using random forest to predict antimicrobial minimum inhibitory concentrations of nontyphoidal *Salmonella* in Taiwan

**DOI:** 10.1186/s13567-023-01141-5

**Published:** 2023-02-06

**Authors:** Chia-Chi Wang, Yu-Ting Hung, Che-Yu Chou, Shih-Ling Hsuan, Zeng-Weng Chen, Pei-Yu Chang, Tong-Rong Jan, Chun-Wei Tung

**Affiliations:** 1 Department and Graduate Institute of Veterinary Medicine, School of Veterinary Medicine, National Taiwan University, Taipei, 106 Taiwan; 2 Animal Technology Laboratories, Agricultural Technology Research Institute, Hsinchu City, 300 Taiwan; 3 Graduate Institute of Veterinary Pathobiology, College of Veterinary Medicine, National Chung Hsing University, Taichung, 402 Taiwan; 4 Graduate Institute of Data Science, College of Management, Taipei Medical University, Taipei, 106 Taiwan; 5 Institute of Biotechnology and Pharmaceutical Research, National Health Research Institutes, Miaoli County, 350 Taiwan

**Keywords:** Antimicrobial resistance, *Salmonella*, minimum inhibitory concentrations, machine learning, k-mer, whole-genome sequencing

## Abstract

Antimicrobial resistance (AMR) is a global health issue and surveillance of AMR can be useful for understanding AMR trends and planning intervention strategies. *Salmonella*, widely distributed in food-producing animals, has been considered the first priority for inclusion in the AMR surveillance program by the World Health Organization (WHO). Recent advances in rapid and affordable whole-genome sequencing (WGS) techniques lead to the emergence of WGS as a one-stop test to predict the antimicrobial susceptibility. Since the variation of sequencing and minimum inhibitory concentration (MIC) measurement methods could result in different results, this study aimed to develop WGS-based random forest models for predicting MIC values of 24 drugs using data generated from the same laboratories in Taiwan. The WGS data have been transformed as a feature vector of 10-mers for machine learning. Based on rigorous validation and independent tests, a good performance was obtained with an average mean absolute error (MAE) less than 1 for both validation and independent test. Feature selection was then applied to identify top-ranked 10-mers that can further improve the prediction performance. For surveillance purposes, the genome sequence-based machine learning methods could be utilized to monitor the difference between predicted and experimental MIC, where a large difference might be worthy of investigation on the emerging genomic determinants.

**Supplementary Information:**

The online version contains supplementary material available at 10.1186/s13567-023-01141-5.

## Introduction

*Salmonella*, belonging to the Enterobacteriaceae family, is a gram-negative rods bacillus. They are widely distributed in animals and are prevalent in food-producing animals, including cattle, porcine, and poultry [1]. *Salmonella* is one of the major food-borne zoonotic pathogens causing approximately 93.8 million global infections with 155 000 deaths per year [2]. The symptoms of salmonellosis are generally mild; however, the severity of the disease depends on the serotypes of *Salmonella* and host factors [3, 4]. The World Health Organization (WHO) estimates that *Salmonella* is one of the four key causes of global diarrhoeal diseases. *Salmonella* has been considered the first priority for inclusion in a program of integrated surveillance of antimicrobial resistance (AMR) in foodborne pathogens by the WHO [5]. In Taiwan, *Salmonella* isolated from healthy poultry and swine is one of the major detected pathogens in our national AMR surveillance program from 2017.

AMR poses a major health problem worldwide. It was estimated that 4.95 million deaths were associated with bacterial AMR in 2019 [6]. Surveillance programs at multidisciplinary level are vital for better understanding of AMR and minimizing the emergence of AMR [7]. Recent advances in fast and affordable whole-genome sequencing technologies have revolutionized microbial surveillance [8]. Whole-genome sequence-based surveillance enabled the detection of multidrug-resistance (MDR) and can be a replacement for phenotypic tests [9–11]. While various online tools and databases are available for AMR detection and surveillance [8], the conventional methods are based on the search of AMR genes using a curated knowledge base such as the Comprehensive Antibiotic Resistance Database (CARD) [12] and ResFinder [13]. However, a knowledge gap was found that may impede the adoption of the conventional methods [10].

Machine learning algorithms are emerging tools for identifying AMR. The genomic sequences can be encoded as feature vectors for training prediction models of AMR. In contrast to the conventional methods relying on sequence comparison to a database, the machine learning methods learn patterns of AMR from training dataset without the issue of knowledge gap and usually provide superior performance over the conventional methods [14, 15]. The machine learning methods and software for AMR detection have been comprehensively reviewed [14]. The methods can be generally classified into qualitative and quantitative methods. The qualitative methods predict AMR based on a predefined dataset of susceptible and resistant isolates. Since the breakpoints for defining AMR may change, retraining of the qualitative methods will be required. Furthermore, the classification of isolates with minimum inhibitory concentration (MIC) near the breakpoint could be unreliable and the isolates were often excluded from the development of qualitative methods [15]. In contrast, the quantitative methods directly predict the MIC values that provide a more flexible option for interpreting the prediction results [16, 17].

Since an MIC variation of up to two two-fold dilutions across laboratories was observed [15, 18, 19], the development and deployment of machine learning algorithms for MIC prediction can benefit from a dataset with well-controlled experimental conditions. In this study, random forest models were developed for AMR prediction of nontyphoidal *Salmonella*. Random forest is a popular ensemble tree-based algorithm consisting of multiple trees, each learned from different bagging samples. The average of predicted values from all trees will be the final predicted result. It is robust even for a small dataset and capable of dealing with high-dimensionality [20], that is suitable for the present work. The sequencing data and MIC measurement for 24 drugs were all generated from the antimicrobial surveillance program supported by the Bureau of Animal and Plant Health Inspection and Quarantine in Taiwan using the same protocols and conducted in the same laboratories. The robust prediction on new isolates not involved in developing the models showed the effectiveness of the models.

## Materials and methods

### Dataset

The WGS and MIC data were obtained based upon works supported by the Council of Agriculture, Executive Yuan, Taiwan, ROC, under grant numbers 106AS-9.12.1-BQ-B1, 107AS-8.9.1-BQ-B1, 108AS-8.8.1-BQ-B1, 109AS-8.8.1-BQ-B1,110AS-5.6.1-BQ-B1, and 111AS-5.6.1-BQ-B1. The *Salmonella* strains were isolated from fecal samples collected randomly from healthy poultry and swine in slaughterhouses in Taiwan. A total of 321 *Salmonella* isolates collected before 2020 were utilized for model training and validation. For each drug, the associated isolates were divided into a training and a validation dataset in a ratio of 8:2. Additional 16 *Salmonella* isolates collected in 2020 were utilized as independent test dataset for assessing the prediction performance of the developed model. The WGS reads were generated using an Illumina MiSeq sequencer (Illumina^®^, San Diego, CA, USA) with paired-end 150 bp sequencing. The reads were trimmed at a Phred quality score of Q30 using Trimmomatic [21], and were de novo assembled using Unicycler with an Illumina-only assembly pipeline [22]. Parameters for genomes assembling include a minimum length of 75 bp. The genome assemblies were used for further analysis.

The final genome assemblies were checked by the quality metrics of the depth of coverage, total read length, N50 and number of contigs for its contiguity. Genome completeness was measured by alignment search of expected gene content and reference genome in Salmonella In Silico Typing Resource (SISTR) database. We followed the quality assessment recommended by the EU Reference Laboratory for antimicrobial resistance (EURL-AR) [23] and revised the quality standard based on our previous experience in strain identification, subtyping, and phylogenetic analysis. The good quality was set at over 50-fold depth of coverage, 4.5–5.5 Mb of total read length, over 20 000 bp of N50 and less than 1000 contigs. If the genome did not reach the quality standard as mentioned above, it was regarded as low quality. Low-quality genomes were removed from all further analysis and sequencing was carried out again.

The MICs of 24 antimicrobial agents, including amoxicillin, ampicillin, azithromycin, cefotaxime, cefoxitin, ceftazidime, ceftiofur, ceftriaxone, chloramphenicol, ciprofloxacin, colistin, enrofloxacin, ertapenem, florfenicol, gentamicin, kanamycin, meropenem, nalidixic acid, oxytetracycline, streptomycin, sulfonamide, tetracycline, tigecycline, and trimethoprim for *Salmonella* isolates were determined by broth microdilution method in accordance with the guideline of the Clinical and Laboratory Standards Institute (CLSI, USA) [24]. The antimicrobial agents were tested at two-fold dilution series with a maximum concentration from 64 to 1024 μg/mL. There are some isolate-drug pairs without MIC evaluation leading to a total number of 4924 and 1246 isolate-drug pairs for model training and validation, respectively. For the independent test dataset, only MICs for 11 drugs were evaluated for the 16 isolates resulting in 176 isolate-drug pairs (shown in Table 1). The breakpoints of CLSI (2021) for the 24 drugs were utilized for classifying susceptible and resistant isolates. The log_2_-transformed MIC (log_2_MIC) values were utilized for following analysis. Detailed numbers of the datasets were shown in Table 2.

**Table 1:** **The numbers of susceptible and resistant isolates based on the CLSI breakpoints (2021) in the independent test dataset for 11 drugs.**

Drugs	Resistant	Susceptible	log_2_MIC (Min., Q1, Q2, Q3, Max.)
Amoxicillin	15	1	(0, 6, 8, 8,8)
Ceftiofur	0	16	(0, 0, 1, 2, 4)
Chloramphenicol	13	3	(3, 7.75, 8, 8, 8)
Ciprofloxacin	1	15	(−2, −1.25, −1, −1, 3)
Colistin	1	15	(−1,−3, −1.5, 1, 2)
Enrofloxacin	0	16	(−1, 0, 0, 0, 1)
Florfenicol	12	4	(4, 5.5, 8, 8, 8)
Gentamicin	1	15	(−1, −1, 0, 1, 6)
Kanamycin	9	7	(2, 4, 8, 8, 8)
Nalidixic acid	7	9	(3, 4, 4, 5.75, 8)
Oxytetracycline	14	2	(3, 8, 8, 8, 8)

**Table 2:** **The numbers of susceptible and resistant isolates in the training and validation dataset and clinical breakpoints for 24 drugs**.

Drugs	Training	Resistant	Susceptible	log_2_MIC (Min., Q1, Q2, Q3, Max.)	Validation	Resistant	Susceptible	log_2_MIC (Min., Q1, Q2, Q3, Max.)	Clinical Breakpoint (μg/mL)
Amoxicillin	256	215	41	(−1, 8, 8, 8, 8)	65	54	11	(−1, 8, 8, 8, 8)	32
Ampicillin	167	135	32	(0, 8, 8, 8, 8)	42	33	9	(0, 8, 8, 8, 8)	32
Azithromycin	146	21	125	(1, 1, 1.5, 2, 8)	37	5	32	(1, 1, 2, 3, 8)	32
Cefotaxime	167	42	125	(−3, −3, −2,1, 6)	42	10	32	(−3, −3, −2, 1.25, 6)	4
Cefoxitin	146	46	100	(1, 2, 3, 6, 8)	37	11	26	(−3, 2, 3, 7, 8)	32
Ceftazidime	167	27	140	(−1, −1, 0, 1, 8)	42	7	35	(−1, 0, 0, 1, 8)	16
Ceftiofur	256	23	233	(−1, 0, 0, 1,8)	65	6	59	(−1, 0, 1, 1, 8)	32
Ceftriaxone	167	27	140	(−3, −3, −3, −2, 6)	42	6	36	(−3, −3, −2, −1.75, 4)	4
Chloramphenicol	256	178	78	(1, 3, 8, 8, 8)	65	45	20	(1, 4, 8, 8, 8)	32
Ciprofloxacin	256	27	229	(−7, −5, −3, −2, 4)	65	7	58	(−7, −3, −2, −1, 6)	1
Colistin	256	44	212	(−3, −3, −1, 1, 3)	65	12	53	(−3, − 3, − 1, 1, 6)	4
Enrofloxacin	256	2	254	(−1, −1, −1, −1, 5)	65	1	64	(−1, −1, −1, 0, 7)	32
Ertapenem	146	14	132	(−3, −3, −3, −3, 6)	37	3	34	(−3, −3, −3, −3, 6)	2
Florfenicol	256	161	95	(1, 3, 6, 8, 8)	65	41	24	(1, 3, 7, 8, 8)	32
Gentamicin	256	34	222	(−1, −1, −1, 1, 8)	65	9	56	(−1, −1, −1, 1, 8)	16
Kanamycin	256	73	183	(0, 1, 2, 8, 8)	65	19	46	(0, 1, 2, 8, 8)	64
Meropenem	167	1	166	(−3, −3, −3, −3, 6)	42	0	42	(−3, −3, −3, −3, −2)	4
Nalidixic Acid	256	102	154	(1, 2, 4, 8, 8)	65	26	39	(1, 2, 4, 8, 8)	32
Oxytetracycline	256	215	41	(0, 7, 8, 8, 8)	65	54	11	(0, 7, 8, 8, 8)	32
Streptomycin	167	122	45	(1, 4, 5, 8, 8)	42	30	12	(1, 4.75, 5, 8, 8)	32
Sulfonamide	167	119	48	(5, 7, 10, 10, 12)	42	29	13	(3, 9.75, 10, 10, 10)	512
Tetracycline	167	135	32	(−1, 6, 7, 8, 8)	42	34	8	(−1, 6, 7, 8, 8)	16
Tigecycline	167	0	167	(−3, −3, −2, −1, 0)	42	0	42	(−3, −2, −2, −1, −1)	4
Trimethoprim	167	115	52	(−1, −1, 8, 8, 8)	42	29	13	(−1, 6.25, 8, 8, 8)	16

### Model development and feature selection

In this study, 10-mer features extracted from the genome sequences of isolates were utilized for machine learning. The k-mer counting (KMC) program [25] was applied to calculate the 10-mer frequencies as features. Theoretically, there will be 4^10^ = 1 048 576 features. After removing the 10-mers not found in our dataset, the total number of features based on the 321 isolates of the training and validation datasets is 524 301. Please note that the test dataset might have additional 10-mer features not found in the training and validation dataset, but those 10-mers were ignored in this experiment. The command utilized for calculating 10-mer counts is “kmc -k 10 -fm -ci1 -cs1677215 input output temp”.

The random forest algorithm was applied to train a prediction model for log_2_MIC. The random forest algorithm has been shown to be effective for predicting AMR [26]. Random forest is an ensemble of *n* decision trees trained on bootstrap samples and *m* randomly selected features. The prediction results were the average of the predictions from the tree ensembles, where the decimals were rounded off. The number of randomly selected features for tree building was set to a default value of *m* = 724 that is the square root of the total feature number. The parameter of *n* was tuned based on out-of-bag (OOB) MAE. The OOB error is a method to estimate predictive performance by applying the model to predict OOB samples that were not involved in the development of a tree. In this study, we considered the *n* ∈ {100, 200, 500, 700, 1000, 1500}. The random seed was set to 0 for reproducibility.

For feature selection, the built-in feature importance function was utilized to rank the features for their importance. Subsequently, the top *k* features were adopted for model training and OOB error evaluation, where *k* ∈ {100, 200,…, 2000}. The scikit-learn 0.23.1 library and python 3.7 programming language were utilized to implement the random forest regressor.

### Performance measurement

The present work aims to predict the MIC value for isolates using genome sequence, therefore the main indicator for measuring the performance of models is the mean absolute error (MAE). As for the classification results based on clinical breakpoints, accuracy, sensitivity, specificity and precision were utilized for evaluating model performance as shown in the following:

1 $$\mathrm{Accuracy}= \frac{TP+TN}{N}$$

2 $$\mathrm{Sensitivity}= \frac{TP}{TP+FN}$$

3 $$\mathrm{Specificity}=\frac{TN}{TN+FP}$$

4 $$\mathrm{Precision}=\frac{TP}{TP+FP}$$

where *N*, *TP*, *TN*, *FP*, and *FN* represent the total number of samples, true positives, true negatives, false positives, and false negatives, respectively.

## Results

### Model development, validation and independent test

**Figure 1:**
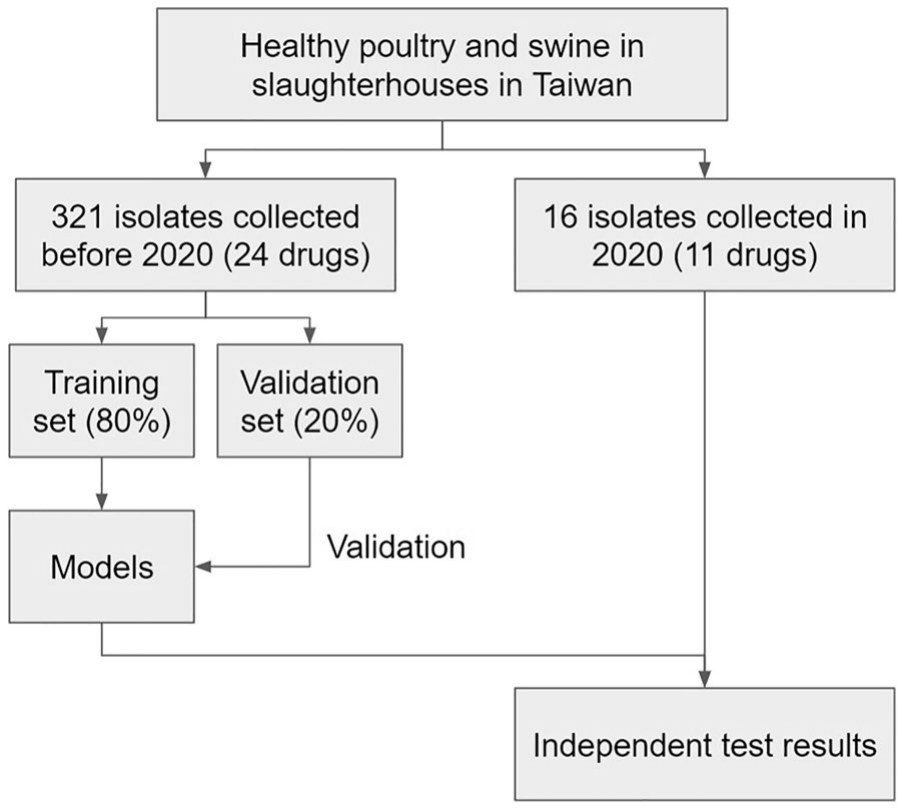
**System flow of the present study**.

**Figure 2:**
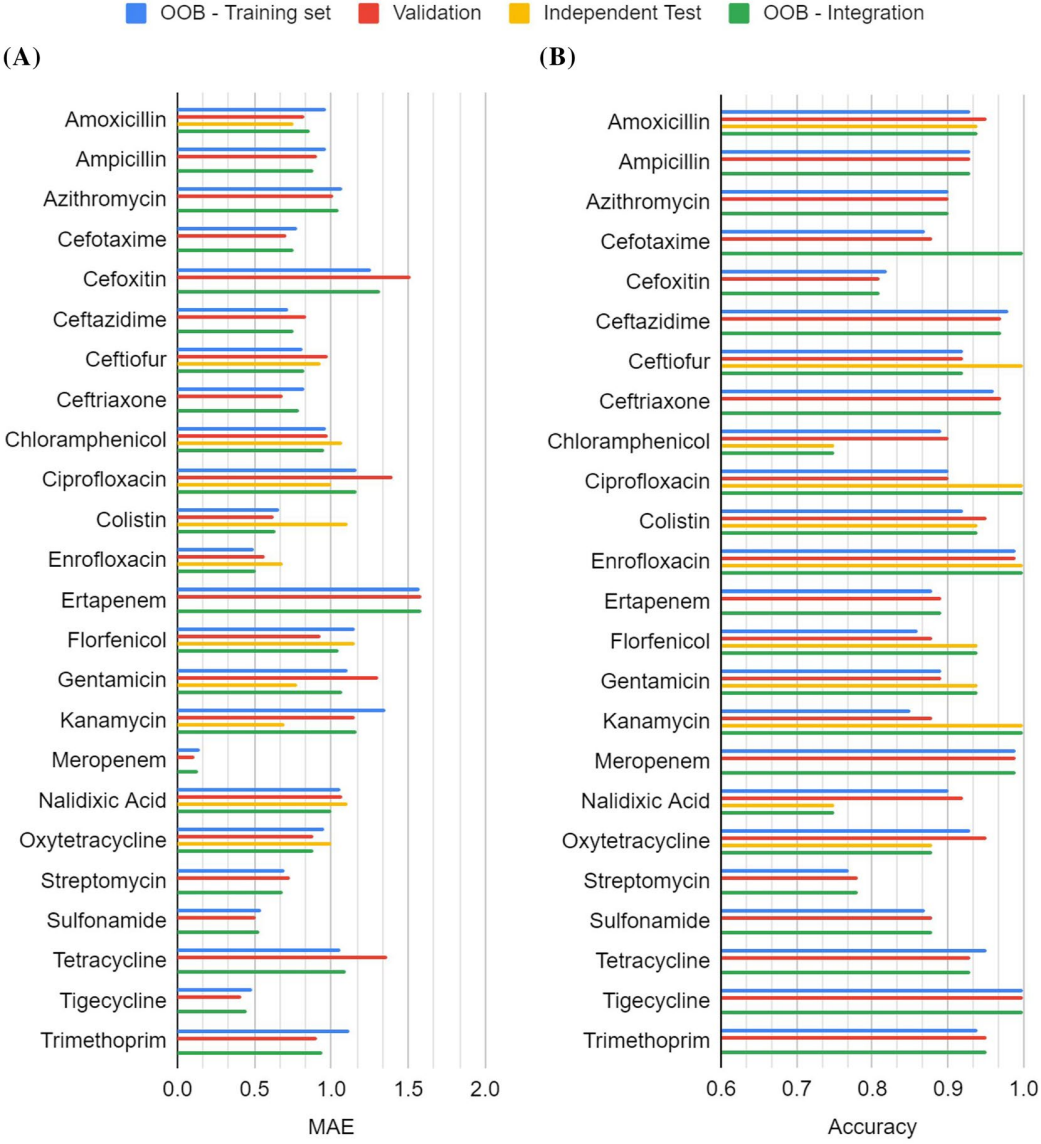
**Performance comparison for 24 drugs and four datasets of training, validation, test and integrated datasets. **OOB out-of-bag, *MAE* mean absolute error.

For model development, parameter tuning was conducted using the training dataset and the tuned parameter was then utilized for training the final prediction models. The system flow of this study is shown in Figure 1. A total of 24 models were developed for predicting the MICs of 24 drugs. The tree number of *n* = 1000 gave the best performance on OOB samples for 24 drugs with an average MAE of 0.916 (Figure 2A). The MAE ranging from 0.916 (*n* = 1000) to 0.927 (*n* = 100) for various numbers of trees indicates a small effect of the parameter of tree number on the MAE performance. The MAE value of less than 1 means the prediction will generally fall within a two-fold dilution range. When taking the breakpoint into consideration, the random forest models are able to distinguish susceptible and resistant isolates with an average accuracy of 91% (Figure 2B). Detailed OOB performance was shown in Additional file 1. The drugs meropenem, tigecycline, and enrofloxacin are associated with the lowest MAEs of less than 0.6. In contrast, ertapenem, kanamycin, and cefoxitin are associated with the worst MAEs of greater than 1.2. To have a better insight, the predictions were further evaluated by using measurements of sensitivity, specificity and precision. Please note that some drugs are associated with only a few resistant isolates that are expected to have low sensitivity. There are 11 drugs with a sensitivity higher than 0.8. Since there is no resistant isolate for tigecycline in the dataset, sensitivity was not calculated. High specificity was obtained for all drugs except for streptomycin with a specificity of less than 0.69. High precision of greater than or equal to 0.8 was obtained for 18 drugs. For three drugs of enrofloxacin, meropenem and tigecycline, all isolates were predicted to have MIC values less than the breakpoints and therefore there are no calculated precision values.

To further evaluate the 24 models, the model performance on the validation dataset was shown in Figure 2 and Additional file 2. Please note that the validation dataset was not involved in the model training, therefore it represents a test on unseen isolates. Overall, similar average values of MAEs and accuracies of 0.92 and 0.92 were obtained for the validation dataset, respectively. A total of 11, 23 and 18 drugs are associated with a performance value greater than or equal to 0.8 in terms of sensitivity, specificity and precision, respectively. The performance measures based on OOB and validation dataset are very similar and are therefore considered less overfitting issues. Four drugs of meropenem, tigecycline, enrofloxacin, and sulfonamide are associated with the lowest MAEs of less than 0.6. Five drugs of ertapenem, cefoxitin, ciprofloxacin, tetracycline, and gentamicin are associated with worst MAEs of greater than 1.2.

While the models provide good performance on OOB and validation datasets, an additional dataset collected in 2020 was utilized to independently test the models to simulate the application of the models for AMR surveillance. The MIC values of 176 unseen isolate-drug pairs were predicted based on the above-mentioned models. As shown in Figure 2, The average MAE and accuracy of 0.94 and 0.92, respectively, are similar to the results obtained from OOB and validation showing no overfitting problems. Detailed information is shown in Additional file 3.

### Performance improvement by enlarging dataset

While the developed prediction models gave a good prediction of MIC values for unseen isolates, the training dataset is relatively small. Since dataset size is a critical factor for developing machine learning models, the continuous integration of newly sequenced and phenotyped isolates into the training dataset could benefit the models. Therefore, this study evaluated the performance change made by enlarging the training dataset. An integrated training dataset was developed by integrating the original training, validation and test datasets. The integration resulted in a dataset of 6346 isolate-drug pairs that were utilized to train new models and evaluate the corresponding OOB errors. As expected, the integration of 1422 isolate-drug pairs improved the average OOB MAE and accuracy by 3.05% and 1.01% with values of 0.88 and 0.92, respectively. A comparison of the OOB performance using the training and integrated datasets is shown in Figure 2. Detailed performance was shown in Additional file 4. The largest improvement in MAE was made for kanamycin, trimethoprim, and amoxicillin with 0.19, 0.18 and 0.11 improvements, respectively. As for accuracy, 15%, 13% and 10% improvement was obtained for kanamycin, cefotaxime, and ciprofloxacin, respectively. Future integration of more isolate-drug pairs could further improve the performance.

### Top-ranked 10-mers as predictive features

The developed models are predictive, however, the high-dimensional feature vector could interfere with the performance of the applied machine learning algorithm and slow down the execution time. The top-*k* features ranked by using the built-in feature importance function estimator of random forest with the lowest OOB and MAE values were identified for each drug. Random forest algorithms were then applied to develop prediction models using the training dataset and top-*k* features. Selected top-ranked 10-mers for each drug are shown in Additional file 5. As shown in Additional file 6, the average MAE and accuracy of 0.72 and 0.93, respectively, were obtained that were much better than the models utilizing all 10-mer features. None of the drugs has an MAE value greater than 1.2. Four drugs of enrofloxacin, meropenem, sulfonamide and tigecycline are associated with low MAE values of less than 0.6. When applying the top-10 models to the validation dataset, the average MAE and accuracy were 0.81 and 0.93, respectively (Additional file 7). Both results suggested that the top-ranked 10-mers are essential predictive features for MIC prediction.

**Figure 3:**
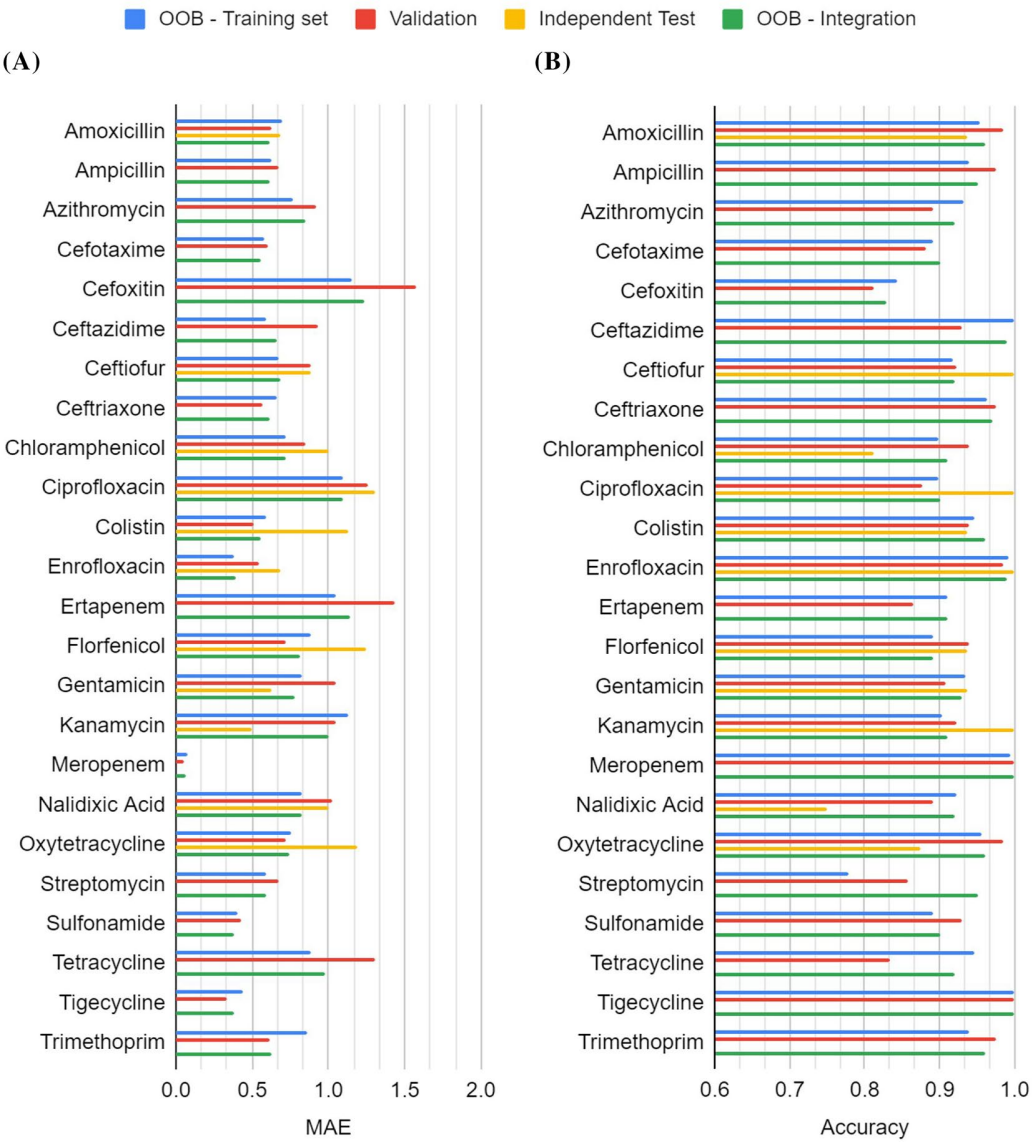
**Performance comparison for 24 drugs and four datasets of training, validation, test and integrated datasets using top-ranked 10-mers.** *OOB* out-of-bag, *MAE* mean absolute error.

**Figure 4:**
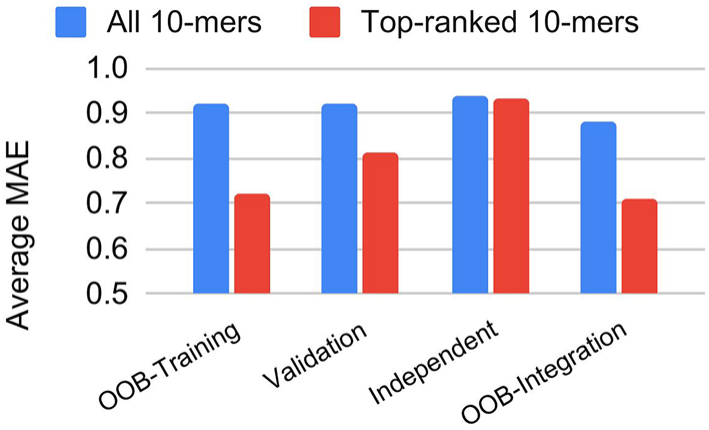
**Comparison of MIC prediction based on all k-mers and top-ranked k-mers**.

An independent test on the additional dataset consisting of 176 unseen isolate-drug pairs collected in 2020 showed slightly improved performance (1%). The average MAE and accuracy of the model using the top-ranked 10-mers are 0.93 and 0.93, respectively. Detailed information is shown in Additional file 8. Compared to the large improvement on the validation dataset, it is unexpected that only a small improvement was made on the independent test dataset. As shown in Figure 3A, the independent test performance of MAE for colistin, florfenicol and oxytetracycline are much worse than those observed in OOB and validation datasets. In contrast, chloramphenicol and nalidixic acid showed much worse accuracies. Since there are only 16 isolates per drug, the bias in performance may be introduced by the small dataset. In addition, gene mutation may result in different 10-mer profiles that may not be captured by the model. To incorporate all available information for developing final models, the integrated dataset of training validation and independent test datasets was utilized to train the final models using the selected top-ranked 10-mers. Detailed information was shown in Additional file 9. The average MAE and accuracy of the model using the top-ranked 10-mers are 0.71 and 0.94, respectively. Altogether, as shown in Figure 4, the top-ranked 10-mers and integration of additional datasets are useful for improving the prediction of MIC.

### Comparison to existing methods

To provide a comparison of the developed method and existing methods, a publicly available machine learning-based tool [16] trained on a public database of PATRIC [27] and a knowledge-based method of ResFinder were applied to predict the samples of the independent test dataset. The machine learning-based tool utilizing XGBoost algorithm was reported to achieve high accuracy for predicting MICs for 15 antibiotics. However, the application of the models for predicting samples of the independent test dataset showed relatively low performance with an average MAE of 2.929 and accuracy of 65%, respectively. Note that some of the antibiotics in the independent test dataset were not covered by the XGBoost models and only amoxicillin, ceftiofur, chloramphenicol, ciprofloxacin, gentamicin, kanamycin, and nalidixic acid were considered in this comparison. For the ResFinder 4.1, the assembled contigs were submitted to the web server at with default parameters. The average accuracy for predicting the samples of the independent test dataset is 75% for amoxicillin, ceftiofur, chloramphenicol, ciprofloxacin, colistin, florfenicol, gentamicin, kanamycin, and nalidixic acid. Detailed performances for the XGBoost-based model and ResFinder are available as Additional files 10 and 11. Both methods did not provide satisfactory performance and stress out the importance of the present work.

## Discussion

AMR is a global health issue and surveillance of AMR can be useful for understanding AMR trends and planning intervention strategies. However, traditional antimicrobial susceptibility testing is time-consuming and labor-intensive. Modern artificial intelligence methods are capable of capturing the patterns hidden in the dataset generated by previous experiments and applying the patterns for predicting unseen samples. As the inter-laboratory variation of MIC measurement can vary up to two two-fold dilutions, the publicly available tool [16] trained on a public database of PATRIC [27] did not produce good prediction results in our datasets. Also, the knowledge-based method of ResFinder did not provide satisfactory performance. Considering the possible knowledge gap and variation of sequencing and MIC measurement methods, it is desirable to develop genome sequence prediction models based on the data produced using the same protocols by the same laboratories in Taiwan.

In this study, genome sequence-based random forest models were developed for predicting MIC values of 24 drugs with rigorous validation and testing using three datasets. The sequence features were represented as a large number of 10-mers and a good performance of an average MAE less than 1 was obtained from the models. The performance comparison of top-ranked 10-mers and all 10-mers highlighted the importance of feature selection. The top-ranked 10-mers can be further mapped to the genome for identifying genes relevant to antimicrobial susceptibility.

The proposed random forest algorithm is a non-linear learning method whose prediction is derived from complex 10-mer-based rules of an ensemble of decision trees. Since AMR can involve multiple genes simultaneously, the proposed method can provide better performance than traditional AMR gene-based methods. However, the identified 10-mers may not be directly linked to a specific phenotype making the interpretation of the associations difficult. The tradeoff between interpretability and prediction performance is a well-known issue. Machine learning methods can provide good performance [28], while the traditional AMR gene identification tool provides good interpretability. Other tools such as DBGWAS [29], while not designed to build predictors for maximizing the prediction performance of phenotypes, may also be utilized to study the associations of k-mers and phenotypes. Furthermore, while out of the scope of this study, as the phylogenetic information can benefit the genome-wide association study [30], the information may be further engineered as new features and evaluated for its contribution to MIC prediction.

As the utilized dataset was obtained from a regular surveillance program on healthy poultry and swine, the data imbalanced issues were expected and that may not hamper the utilization of the model due to the nature of the model for predicting MIC values rather than susceptibility. However, prediction performance of susceptibility classification should be interpreted with care. For example, enrofloxacin was associated with only two, one and zero resistant isolates in the training, validation and test dataset, respectively. The predictor learned more from the MIC distribution of the majority class of susceptible isolates and predicted the susceptible isolates well. As a result, the accuracy for enrofloxacin is notably high, and this can primarily be attributed to the majority class.

For surveillance purposes, the genome sequence-based machine learning methods could be utilized to monitor the difference between predicted and experimental MIC, where a large difference might be worthy of investigation on the emerging genomic determinants. This study presented a successful machine learning-based MIC prediction method utilizing genomic and phenotypic data obtained from surveillance programs in Taiwan. The incorporation of future data is expected to further improve the prediction performance of the models.

## Supplementary Information

**Additional file 1. The OOB validation performance using the training dataset.**

**Additional file 2. The validation performance using the validation dataset.**

**Additional file 3. The test performance using the independent test dataset.**

**Additional file 4. The OOB validation performance using the integrated dataset.**

**Additional file 5. The top-ranked k-mers for each drug.**

**Additional file 6. The OOB validation performance using the training dataset and top k-mers.**

**Additional file 7. The validation performance using the validation dataset and top k-mers.**

**Additional file 8. The test performance using the independent test dataset and top k-mers.**

**Additional file 9. The OOB performance using the integrated dataset and top k-mers.**

**Additional file 10. Prediction performance of an XGBoost-based model [16] using the independent test dataset.**

**Additional file 11. Prediction performance of ResFinder using the independent test dataset.**

## Data Availability

The data that support the findings of this study are available from the Bureau of Animal and Plant Health Inspection and Quarantine of Taiwan but restrictions apply to the availability of these data, which were used under the contract numbers 109AS-8.8.1-BQ-B1, 110AS-5.6.1-BQ-B1 and 111AS-5.6.1-BQ-B1 for the current study, and so are not publicly available. Data are however available from the authors upon reasonable request and with permission of the Bureau of Animal and Plant Health Inspection and Quarantine of Taiwan.
